# New York—American Veterinary College

**Published:** 1902-08

**Authors:** 


### COMMENCEMENT EXERCISES.

### NEW YORK—AMERICAN VETERINARY COLLEGE.

The annual commencement exercises of this school were held in conjunction with the School of Law, the University and Bellevue Hospital Medical College, the Graduate School, the School of Pedagogy, the School of Commerce, Accounts and Finance (all departments of New York University), on Thursday evening, June 5th, at the Metropolitan Opera House. The exercises attending the closing of the University and the University and College of Applied Sciences had been proceeding at University Heights since May 31st. The Opera House was packed, and it was estimated that a thousand were unable to obtain entrance.

The following passed satisfactory examinations before the Faculty, and all those who had received the requisite counts before the Regents received the diploma of the college. The remainder will be granted parchments when their preliminary counts are obtained: Oscar Barnett, Jr., Newark, N. J.; Louis Janeway Belloff, New Brunswick, N. J.; George A. Hazel, Brooklyn, N. Y.; Robert Anderson McAuslin, Brooklyn, N. Y.; Warren J. Palmer, New York City; James Lee Shorey, V.S., Hoosick Falls, N. Y.; James L. Wells, Good Ground, L. I., and Roland T. King, Brooklyn, N. Y. Dr. Joseph L. Serling, New York, who passed the Faculty last year and received the requisite counts since, was awarded his diploma.

Robert A. McAuslin passed the best general examination, and received the Faculty gold medal.

Warren J. Palmer passed the second best general examination, and was awarded the Alumni prize of standard veterinary works.

Dr. Palmer was also the recipient of the practical prize of a case of surgical instruments, for the best practical examination passed before a board of veterinarians appointed by the faculty.

